# Periosteal Ewing's Sarcoma: Report of Two New Cases and Review of the Literature

**DOI:** 10.1080/13577149977695

**Published:** 1999-09

**Authors:** Yehuda Kollender, Shay Shabat, Alexander Nirkin, Josephine Issakov, Gideon Flusser, Ofer Merimsky, Isaac Meller

**Affiliations:** 1 The National Unit of Orthopedic Oncology The Tel-Aviv Sourasky Medical Center Tel Aviv Israel; 2 The Unit of Bone and Soft Tissue Pathology The Tel-Aviv Sourasky Medical Center Israel; 3 The Department of Radiology Tel-Aviv Medical School Israel; 4 The Department of Oncology Tel-Aviv Medical School Israel

## Abstract

*Background.* The origin of Ewing's sarcoma in a periosteal location is rare and not clearly documented. Other malignant bone tumors appear to have a somewhat better prognosis when confined between periosteum and bone. Is it the same for periosteal Ewing's sarcoma?

*Methods.* We describe two new cases and comprehensively review the literature consisting of 18 documented cases since the condition was first described in 1986 (S.M. Bator.*Cancer* 58:1781– 4).

*Results.* Periosteal Ewing's sarcoma differs from the other forms of Ewing's sarcoma in terms of sex predominance, location of tumor, surgical stage at presentation and typical imaging studies. Eighteen out of the 20 patients were reported to be alive with no evidence of disease.

*Conclusions.* It seems that the prognosis of this rare variant of Ewing's sarcoma family of tumors might be better but the small number of cases precludes such a firm conclusion.


*Sarcoma* (1999) 3, 85–88

ORIGINAL ARTICLE

### Periosteal Ewing's sarcoma: report of two new cases and review of the literature

YEHUDA KOLLENDER,[1] SHAY SHABAT,[1] ALEXANDER NIRKIN,[1] JOSEPHINE ISSAKOV,[2] GIDEON FLUSSER,[3] OFER MERIMSKY[4] & ISAAC MELLER[1]

[1]*The National Unit of Orthopedic Oncology,* [2]*The Unit of Bone and Soft Tissue Pathology,* [3]*The Department of Radiology &* [4]*The Department of Oncology, The Tel-Aviv Sourasky Medical Center, and Tel-Aviv Medical School, Israel*

**Abstract**

*Background.* The origin of Ewing's sarcoma in a periosteal location is rare and not clearly documented. Other malignant bone tumors appear to have a somewhat better prognosis when confined between periosteum and bone. Is it the same for periosteal Ewing's sarcoma?

*Methods.* We describe two new cases and comprehensively review the literature consisting of 18 documented cases since the condition was first described in 1986 (S.M. Bator. *Cancer* 58:1781–4).

*Results.* Periosteal Ewing's sarcoma differs from the other forms of Ewing's sarcoma in terms of sex predominance, location of tumor, surgical stage at presentation and typical imaging studies. Eighteen out of the 20 patients were reported to be alive with no evidence of disease.

*Conclusions.* It seems that the prognosis of this rare variant of Ewing's sarcoma family of tumors might be better but the small number of cases precludes such a firm conclusion.

**Key words**: *Ewing's sarcoma, periosteal (periosteum), bone tumors, limb-sparing surgery*

### Introduction

The classification of primary malignant bone tumors is still expanding to include new subtypes based upon the clinical presentation, radiographic features (including modern imaging modalities such as computed tomography (CT), magnetic resonance imaging (MRI), positron emission tomography scan (PET scan) and others,[1–4] anatomical localization and new sophisticated modern cytogenetic and molecular biology techniques.[5–9]

It is obvious that the classical histological/morphological methods of classifying bone tumors are crude and not of sufficient accuracy to distinguish subtypes. Subtyping is most probably of prognostic significance, but might have therapeutic implications. Many think that bone tumors arising in a periosteal location (surface lesions) have a somewhat better prognosis than those arising in the medullary cavity of the same bones.[10–11] Ewing's sarcoma (ES) is one of the best examples of a distinctive tumoral disease which, based upon clinical radiological and cytogenetic parameters,[1,9] which, is considered today to be a group of different diseases. According to the anatomical site today we recognize three subtypes: (a) the intraosseous type, which is the most common;[12] (b) the extraskeletal or soft tissue type (less common);[13–14] and (c) the very rare variant of periosteal location of which only 18 cases are described in the literature so far.[15–19]. The first description of periosteal Ewing's sarcoma (PES) was probably that published in 1956 by Sherman and Soong in a comprehensive radiological review of 111 cases of ES of bone which included a roentgen classification.[20]They described three cases of PES among 12 other cases, which they defined as "cortical Ewing's sarcoma of long bones," but without mentioning the name and obviously based only upon plain X-ray films and classical histological criteria. The first well-established case report of PES was published in 1986 by Bator *et al.*[15] He actually defined PES: ". . . in a periosteal location without extension into either the bone or adjacent soft tissues". Since then four additional papers, describing a total of 18 cases, have been published.[16–19]We add to this list two new cases and review the literature.

### Case reports

(1) A 16-year-old male patient was referred to the Orthopedic Oncology Unit in our center on July 1994. He complained of a growing, large (>10 cm) painful

Correspondence to: Isaac Meller, The National Unit of Orthopedic Oncology, Tel Aviv-Elias Sourasky Medical Center, Tel Aviv, Israel.

1357-714X print/1369-1643 online/99/020085-04 © 1999 Taylor & Francis Ltd mass in the postero-lateral aspect of the distal half of his right thigh, which he had experienced for 3 months. His general condition was good except for low fever for the last few months. Physical examination revealed a tender longitudinal mass along the biceps muscle in the right distal thigh, 12.5 cm in size. No palpable lymph nodes were noted in the groin or other places. Blood tests showed an elevated erythrocyte sedimentation rate (ESR) of 100/120; normal white blood cell (WBC) count; normal alkaline phosphatase (AP) blood levels; and a slight increase of lactate dehydrogenase (LDH) blood levels. Plain X-ray films of the thigh and knee region showed priosteal elevation and thickening. Our differential diagnosis was of an infectious disease, such as primary osteomyelitis, or secondary to a soft tissue process or some form of a malignant surface bone neoplasm. Protocol staging studies included: plain chest X-ray film; total body bone scan; CT of the lesion and chest; and MRI of the lesion. The CT and MRI of the distal femur showed a periosteal/surface lesion, as the medullary canal and the endosteal surface of the distal femur were intact. The systemic bone scan and chest CT were normal. The patient underwent an open incisional biopsy under general anesthesia. The histopathological results indicated a classical ES in a periosteal location. According to AMSTS (Enneking's) surgical staging system the patient was in stage II-B.[21] He received neoadjuvant chemotherapy and, in December 1994, underwent a limb-sparing operation where the distal half of the right femur and knee joint were resected including the lateral hamstring muscles and biopsy scar. The defect was replaced by a modular endoprosthesis. Histopathological evaluation of the specimen showed 100% necrosis and practically no tumor mass was found. He continued the same chemotherapy until July 1995. The area was not irradiated and during the follow-up period of 3 years since then he has been free of disease.

(2) A 27-year-old male patient was referred to our unit on October 1995 because of a painful growing mass in the medial aspect of his right thigh for 2½ months. His general condition was good. Physical examination showed a tender longitudinal lesion in the right mid-medial aspect of his thigh. There was no evidence of palpable lymph nodes in any locations. Blood tests, including ESR, WBC count, AP and LDH, were all normal. Conventional radiograph of the femur showed generalized periosteal thickening, with an area of bulging periosteum and a slight hypodense region within it. A soft tissue component was noted (Fig. 1). Our differential diagnosis was either a soft tissue tumor encroaching upon the bone or a malignant surface bone neoplasm. Staging studies included plain chest X-ray film; total body bone scan; CT of the lesion and chest and MRI of the lesion. As in the previous case, the CT and MRI of the thigh showed a periosteal/surface lesion without any involvement of the medullary canal (Fig. 2). Systemic bone scan and chest CT were normal. On October 1995 he underwent a core needle biopsy. The histopathological result was classical ES in a periosteal location. According to AMSTS the patient was in stage II-B.[21] Neoadjuvant chemotherapy and preoperative radiotherapy (4500 rad) were given. He underwent a limb-sparing procedure where one-half of the mid-femoral shaft and adductor muscles were resected. The defect was reconstructed with an intramedullary nail and autologous bone graft. Histopathological evaluation of the specimen showed 100% necrosis. He continued chemotherapy and post-operative radiotherapy up till February 1997 and during the follow-up period of 1.5 years the patient has remained free of disease.

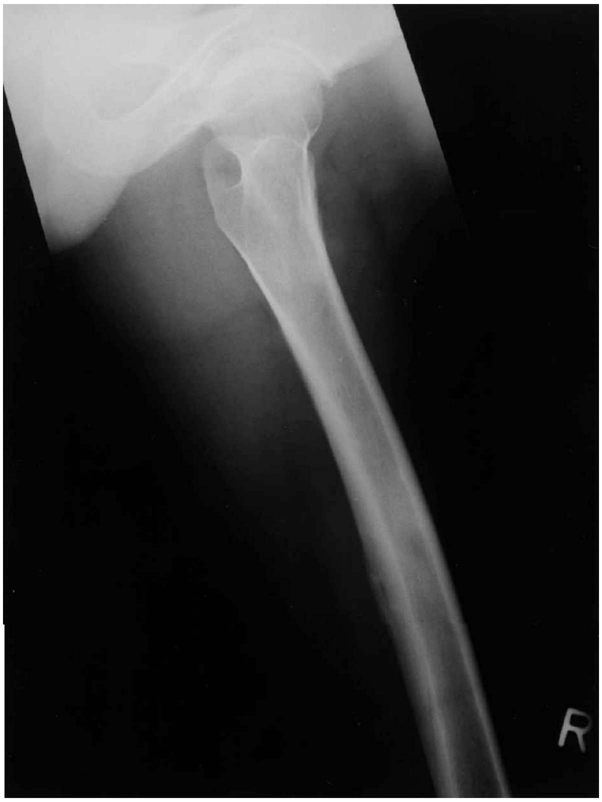

**Fig. 1.** *Conventional radiograph of the femur shows generalized periosteal thickening with an area of bulging periosteum and a slight hypodense region within it. A soft tissue component is noted.*

### Review of the literature (18 cases)

Eighteen cases, described in five papers published from 1986 to 1994,[15–19] together with the two new cases, comprise this survey.

There were 17 males and three females aged between 11 and 30 years. Presenting symptoms were mentioned for 14 of the 20 patients[15,17–19] and included: pain (four patients), a mass (one patient) and a combination of both in the remaining nine patients. Duration of symptoms varied between 10 days and 4 months (mean, 2 months). In 16 cases the tumors were located in the proximal long bones (six in the humerus and 10 in the femur), two in the tibia,

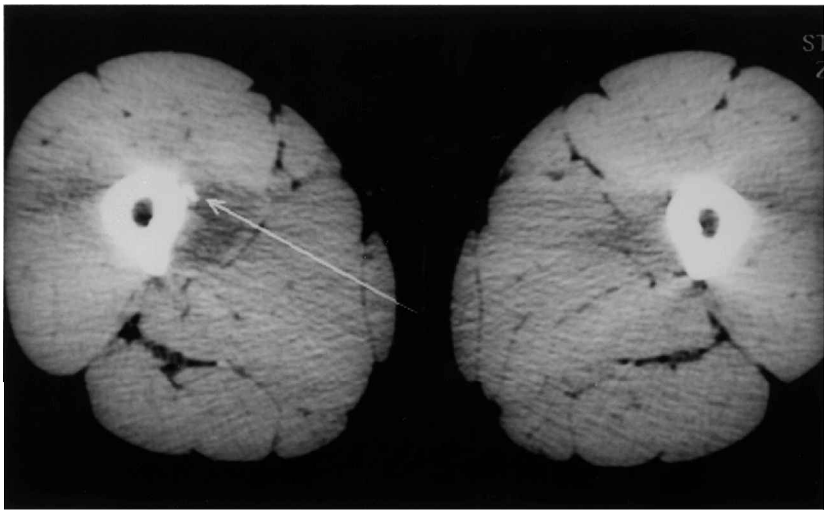

**Fig. 2.** *CT scan of the mid-femur shows a periosteal mass with a soft tissue component. A scalloping of the cortext is seen. The density of the medulla appears normal.*

one in the fibula and one involving the scapula. In 16 cases the tumor was diaphyseal and in three it was metadiapheaseal. All patients were at stage II-B at presentation (according to AMSTS),[21] meaning that no metastases were detected in baseline staging studies.

Non-surgical treatment included chemotherapy for all 20 patients. For 14 patients details were noted about the method by which chemotherapy was given. In 13 of the 14 cases neoadjuvant chemotherapy was given and in one case adjuvant chemotherapy was given. Precise documentation about the timing of radiation therapy was also noted for 14 of the 20 patients. Of these, five patients received external beam radiation therapy (two patients received only preoperative radiation therapy and three patients received combined pre- and post-operative radiation therapy). All of the remaining six patients received radiation therapy, but whether it was pre- or post-operative was not stated. Hence, 11 of the 20 patients received radiotherapy.

Nineteen of the 20 patients had tumors located in long bones (one was at the scapula). Of these 19 patients, 18 underwent limb-sparing surgery and one underwent amputation.

Follow-up periods stated in the papers at publication were between 2 months and 10 years, with a mean of 3 years. For 11 patients follow-up periods of more than 2 years were noted. Two patients were dead, at 1 and 2 years after end of therapy, and the remaining 18 patients were alive with no evidence of disease. The fate of the patients after the publication of the papers is not known to us.

### Discussion

Concerning the clinical presentation and age distribution, no differences between PES and other forms of ES[1,12,22] have been shown. There are differences, however, between PES and the other forms of ES with regard to sex predominance, location of tumor and surgical stage at presentation. A clear male predominance is noted in PES (the male to female ratio is 5.7:1),[15–19] while in medullary ES only a slight male predominance, with a male to female ratio of about 1.5:1, is known[23,24] and there has been no male predominance in extraskeletal ES reported.[13,14].

Medullary[12,23–24] and extraosseous[13,14,25] ES develop in both proximal and distal long bones and axial flat bones. PES shows a predominance in proximal extremities and axial PES is uncommon. Only one case of PES in an axial flat bone (scapula) was reported by Kolar *et al.*[16] The small number of cases precludes a real statistical significance to these clinical observations which show only trends.

In PES, all 20 patients were diagnosed at stage II-B of the disease, with no evidence of distant metastases. In comparison, metastases at presentation occur in about 25% of cases of medullary ES[12,23] and in about 10% of cases of extraskeletal ES.[13]

Imaging studies help to confirm the diagnosis of PES which is defined when there is no tumor invasion of the medullary cavity.[10,11,26–29]. A subperiosteal mass with a periosteal thickening and a Codman triangle are diagnostic.[10,11,26–30] These radiological signs appear both in PES and medullary ES, but PES usually shows a uninterrupted periosteal reaction compared with the 'onion skin' periosteal reaction observed medullary ES.[20] A subperiosteal location and the absence of medullary bone involvement help to distinguish PES from the other types of ES.[3,4,15–17,20,31,32]

Although conventional radiography provides the most useful information for diagnosis and for gauging biological aggressiveness of the tumor, it has some limitations in estimating the extent of intramedullary disease in medullary ES or in soft tissue involvement.[1] Tumor size and the accurate margins between the intramedullary space, the periosteal location and the soft tissue can be adequately determined only by imaging studies such as CT or MRI.[1] The typical picture of PES is of a periosteal tumor which has not invaded the medullary cavity.[15–19] Extraskeletal (soft tissue) ES tumors which grew enough to invade the periosteum will be defined as PES, so there might be an overlap between the two subtypes. Still, the entity of PES is quite well established, and there is a difference between the periosteal form and the soft tissue form in terms of sex, anatomical location in bones and staging at diagnosis.

At histopathological examination, all subtypes of ES, whether medullary, extraskeletal or periosteal, appear the same. In general, ES consists of uniform, small, round or oval highly undifferentiated cells with a pale appearance and scanty cytoplasm. It contains glycogen-positive granules with positive periodic acid–Schiff stain.[12–17,25]

It is not understood why PES seems to have a better prognosis than the other two forms. This observation is similar to that of a better prognosis in periosteal osteosarcoma and periosteal chondrosarcoma than in their medullary counterparts.[10,11] One possible explanation is that the location at the periosteum causes such pain that the patients seek medical help earlier. Another possible explanation can be found in the cytogenetic profile of the patients. None of the 18 patients in the five articles reviewed, together with our own two patients, underwent cytogenetic analysis. The reason for this favorable prognosis may be the latter. It is strongly recommended that such an analysis is performed for PES patients in the future.

After reviewing the literature it seems to us that this rare entity should be considered in the differential diagnosis of the ES family of tumors since there is a possibility that it has a better prognosis than medullary or soft tissue ES.

### References

1 Eggli KD, Quiogue T, Moser RP. Ewing's sarcoma. *Radiat Clin North Am* 1993; 31(2):325–37.
2 Hanna SL, Fletcher BD, Kaste SC, *et al.* Increased confidence of diagnosis of Ewing sarcoma using T2-weighted MR images. *Magn Reson Imaging* 1994; 12(4):559–68.
3 Frouge C, Vanel D, Coffre C, *et al.* The role of magnetic resonance imaging in the evaluation of Ewing sarcoma. *Skelet Radiol* 1988; 17:387–92.
4 Vanel D, Contsso G, Couanet D, *et al.* Computed tomography in the evaluation of 41 cases of Ewing's sarcoma. *Skelet Radiol* 1982; 9:8–13.
5 McManus AP, Gusterson BA, Pinkerton CR, Shipley JM. Diagnosis of Ewing's sarcoma and related tumors by detection of chromosome 22q12 translocations using fluorescence in situ hybridization on tumor touch imprints. *J Pathol.* 1995; 176(2):137–42.
6 Ladanyi M, Lewis R, Jhanwar SC, *et al.* MDM2 and CDK4 gene amplification in Ewing's sarcoma. *J Pathol* 1995; 175(2):211–7.
7 Dierick AM, Langlois M, Van-Oostveldt P, Roels H. The prognostic significance of the DNA content in Ewing's sarcoma: a retrospective cytophotometric and floe cytometric study. *Histopathology* 1993; 23(4):333–9.
8 Taylor C, Patel K, Jones T, *et al.* Diagnosis of Ewing's sarcoma and peripheral neuroectodermal tumor based on the detection of t(11;22) using fluorescence in situ hybridisation. *Br J Cancer* 1993; 67(1):128–33.
9 Stephenson CF, Bridge JA, Sandberg AA. Cytogenetic and pathologic aspects of Ewing's sarcoma and neuroectodermal tumors. *Hum Pathol* 1992; 23(11):1270–7.
10 Bertoni F, Boriani S, Laus M, Campanacci M. Periosteal chondrosarcoma and periosteal osteosarcoma: Two distinct entities. *J Bone Jt Surg [Br]* 1982; 64:370–6.
11 Unni KK, Dahlin DC, Beabout JW. Periosteal osteogenic sarcoma. *Cancer* 1976; 37:2476–85.
12 Wilkins MR, Pritchard DJ, Burgert EO, Unni KK. Ewing's sarcoma of bone: experience with 140 patients. *Cancer* 1986; 58:2551–5.
13 Rud NP, Reiman HM, Pritchard DJ, *et al.* Extraosseous Ewing's sarcoma. A study of 42 cases. *Cancer* 1989; 64:1548–53.
14 Shimada H, Newton WA, Soule EH, *et al.* Pathologic features of extraosseous Ewing's sarcoma: a report from the Intergroup Rhabdomyosarcoma Study. *Hum Pathol* 1988; 19:442–53.
15 Bator SM, Bauer TW, Marks KE, Norris DG. Periosteal Ewing's sarcoma. *Cancer* 1986; 58:1781–4.
16 Kolar J, Zidkova H, Matejovsky Z, *et al.* Periosteal Ewing's sarcoma. *Fortschr Rontgentstr* 1989; 150:179–82.
17 Wuisman P, Roessner A, Blasius S, *et al.* (Sub) Periosteal Ewing's sarcoma of bone. *J Cancer Res Clin Oncol* 1992; 118:72–4.
18 Shapeero LG, Vanel D, Sundaram M, *et al.* Periosteal Ewing's sarcoma. *Radiology* 1994; 191:825–31.
19 Kenan S, Abdelwahab IF, Klein MJ, *et al.* Case report 819: periosteal Ewing's sarcoma of the tibia. *Skelet Radiol* 1994; 23:59–61.
20 Sherman RS, Soong KY. Ewing's sarcoma: its roentgen classification and diagnosis. *Radiology* 1988; 17:387–92.
21 Enneking WF. A system of staging musculoskeletal neoplasms. *Clin Orthop* 1986; 204:9–24.
22 Bacci G, Toni A, Avella M, *et al.* Long term results in 144 localized Ewing's sarcoma patients treated with combined therapy. *Cancer* 1989: 63:1477–86.
23 Kinsella TJ, Miser JS, Waller B, *et al.* Long term follow-up of Ewing's sarcoma of bone treated with combined modality therapy. *Int J Radiat Oncol Biol Phys* 1991; 20:389–95.
24 Nesbit ME, Gehan EA, Burgert EO, *et al.* Multimodal therapy for the management of primary, nonmetastatic Ewing's sarcoma of bone: a long term follow-up of the first intergroup study. *J Clin Oncol* 1990; 8:1664–74.
25 Stauart-Harris R, Wills EJ, Philips J, *et al.* Extraskeletal Ewing's sarcoma: a clinical, morphological and ultrastructural analysis of five cases with a review of the literature. *Eur J Cancer Clin Oncol* 1986; 22:393–400.
26 Nojima T, Unni KK, McLeod RA, Pritchard DJ. Periosteal chondroma and periosteal chondrosarcoma. *Am J Surg Pathol* 1985; 9:666–77.
27 deSantos LA, Murray JA, Finklestein *et al.* The radiographic spectrum of periosteal osteosarcoma. *Radiology* 1978; 127:123–9.
28 Lewis MM, Kenan S, Yabut SM, *et al.* Periosteal chondroma: a report of ten cases and review of the literature. *Clin Orthop Rel Res* 1990; 256:185–92.
29 Abdelwahab IF, Kenan S, Hermann H, *et al.* Periosteal ganglia: CT and MR imaging features. *Radiology* 1993; 188:245–8.
30 Hall RB, Robinson LH, Malawar MM, Duni WK. Periosteal osteosarcoma. *Cancer* 1985; 155:165–71.
31 Rose JS, Hermann G, Mendelson DS, Ambinder EP. Extraskeletal Ewing's sarcoma with computed tomography correlation. *Skelet Radiol* 1983; 9:234–7.
32 O'Keeffe F, Lorigan JG, Wallace S. Radiological features of extraskeletal Ewing's sarcoma. *Br J Radiol* 1990; 63:456–60.
